# Comparison of Count Normalization Methods for Statistical Parametric Mapping Analysis Using a Digital Brain Phantom Obtained from Fluorodeoxyglucose-positron Emission Tomography

**DOI:** 10.22038/AOJNMB.2018.11745

**Published:** 2019

**Authors:** Thet Pe Win, Yoshiyuki Hosokai, Takashi Minagawa, Kenzo Muroi, Kenta Miwa, Ayaka Maruyama, Toshiya Yamaguchi, Kazuto Okano, Khin Moh Moh Htwe, Haruo Saito

**Affiliations:** 1 International University of Health and Welfare, School of Health Sciences, Department of Radiological Sciences, Japan; 2 Tohoku University Graduate School of Medicine, Course of Radiological Technology, Diagnostic Image Analysis, Japan

**Keywords:** Count normalization, FDG- PET, Neurodegenerative disease, SPM analysis, Yakushev method

## Abstract

**Objective(s):**

Alternative normalization methods were proposed to solve the biased information of SPM in the study of neurodegenerative disease. The objective of this study was to determine the most suitable count normalization method for SPM analysis of a neurodegenerative disease based on the results of different count normalization methods applied on a prepared digital phantom similar to one obtained using fluorodeoxyglucose-positron emission tomography (FDG-PET) data of a brain with a known neurodegenerative condition.

**Methods:**

Digital brain phantoms, mimicking mild and intermediate neurodegenerative disease conditions, were prepared from the FDG-PET data of 11 healthy subjects. SPM analysis was performed on these simulations using different count normalization methods.

**Results:**

In the slight-decrease phantom simulation, the Yakushev method correctly visualized wider areas of slightly decreased metabolism with the smallest artifacts of increased metabolism. Other count normalization methods were unable to identify this slightly decreases and produced more artifacts. The intermediate-decreased areas were well visualized by all methods. The areas surrounding the grey matter with the slight decreases were not visualized with the GM and VOI count normalization methods but with the Andersson. The Yakushev method well visualized these areas. Artifacts were present in all methods. When the number of reference area extraction was increased, the Andersson method better-captured the areas with decreased metabolism and reduced the artifacts of increased metabolism. In the Yakushev method, increasing the threshold for the reference area extraction reduced such artifacts.

**Conclusion:**

The Yakushev method is the most suitable count normalization method for the SPM analysis of neurodegenerative disease.

## Introduction

Statistical parametric mapping (SPM) analysis is used as a data analysis tool in functional neuroimaging studies (1–6). It is currently used in clinical settings as a diagnostic aid for cerebral blood flow scintigraphy in Alzheimer’s disease (7) and as an objective index for identifying areas with decreased volume during magnetic resonance imaging (MRI) (8, 9). Statistical dispersion is a major issue in SPM analysis where dispersion may be caused by a variety of different factors. Pretreatment is required to minimize dispersion prior to data analysis if a highly precise SPM analysis is carried out on data with significant dispersion (10). Anatomic normalizations minimizing individual differences in forms and count normalizations conducted on data in which morphological dispersion has been reduced are examples of such pretreatments. Count normalization is the process of setting a standard state (count) for the brain and normalizing to this value (9, 11). The area of the brain least affected by the disease under study is used as a reference area to set the standard value for the rest of the brain and statistical dispersion is reduced through normalization. This allows differences in the state of the brain to be interpreted as changes that are unique to a specific disease. 

Global mean (GM) count normalization is the most commonly used count normalization (5, 6, 12, 13) in SPM. It uses the whole brain (excluding the ventricles and the cerebrospinal fluid outside the brain) as a reference area. Using a wide area as a reference allows the impact of localized changes to be captured and enables the collection of stable values without large variations. It is thus considered to be a superior method. However, if GM count normalization is used for the study of neurodegenerative disease, in which the metabolism of the whole brain is decreased, it carried out the decreased state as the standard leading to underestimation of lowered metabolism and artificial elevations which were inconsistent with clinical symptoms (9, 13-15). For example, Borghammer et al. (9, 11) reported that, based on a meta-analysis of clinical data, brain metabolism decreases with neurodegenerative disease. At the same time, SPM analysis reported hypermetabolism in patients with neurodegenerative disease (1–4). Similar results were reported in studies of schizophrenia (16), depression (17), Alzheimer’s disease (12, 18), etc. Inconsistencies in SPM analyses using GM count normalization are due to the use of areas that are altered by disease as the reference range. To solve this issue, two categories of new count normalization methods have been proposed (9, 13-15): Volume of interest (VOI) count normalization method (9, 14) and Andersson (15) and Yakushev methods (13). The former uses cerebellum and pons as the reference area and the latter use areas with the least functional changes based on statistical analysis as the reference. The Yakushev method is reported as the most superior count normalization method for neurodegenerative disease (9, 11). However, there are no studies examining the impact of different count normalization methods on SPM analysis in detail. 

The objective of this study was to prepare a digital brain phantom similar to one obtained using fluorodeoxyglucose-positron emission tomography (FDG-PET) data of a brain with a known neurodegenerative condition. Then SPM analysis was performed on this phantom using each count normalization method to determine the most suitable one for SPM analysis of a neurodegenerative disease.

## Methods

***Subjects***

In this study, we used FDG-PET data from 11 healthy subjects recruited from the local community through advertisements. The average age ± standard deviation was 62.6 ±4.5 years. They were six males and five females. All subjects (1) were without any history of other neurological and psychiatric diseases, (2) did not exhibit any lesion as detected by a head MRI, (3) had a Clinical Dementia Rating (CDR) score of zero, and (4) had a Mini-Mental State Exam (MMSE) score of 29 or 30. This study was approved by the Tohoku University School of Medicine Ethics Committee and was conducted according to the Declaration of Helsinki. All participants and their families were provided with sufficient information regarding the study and the study was only implemented upon obtaining written consent. 

***PET scan***

We obtained PET scans of the healthy subjects within 4 weeks of neuropsychological testing. Subjects were told to fast 5 h before imaging and were administered an intravenous injection of FDG (185-218 MBq) 1 h prior to the scan. Subsequently, they were allowed to rest in a quiet room while wearing an eye mask until the time of imaging. A Biograph DUO PET/computed tomography (CT) scanner (Siemens Medical System, Inc., USA) was used for PET data collection. The imaging time lasted for 10 min, during which three-dimensional data were collected. Image reconstruction was performed with the ordered subset expectation maximization (OS-EM) method (number of subsets=16, number of iterations=6, Gaussian filter, filter full-width at half maximum (FWHM): 2.0 mm) and a reconstructed image with a matrix size of 256 × 256, a pixel size of 1.33×1.33 mm, and a slice thickness of 2.0 mm was prepared. FWHM for horizontal cross-section reconstruction was 3.38×3.38 mm. A CT scan was used for the collection of attenuation correction data.

***Preparation of the digital brain phantom***

***Software***

 We prepared a digital brain phantom using the following software:

MATLAB:7.11.0 R2010b

SPM8 (Welcome Department of Imaging Neuroscience, London, UK) 

Prominence Processor Ver.3.1 (Nihon Medi-Physics Co., Ltd., Tokyo)

MRIcro (Neuropsychology Lab, Columbia, SC, USA) 

fMRIstat (McGill University, Quebec, Canada, http://www.math.mcgill.ca/keith/fmristat) 

***Construction of digital brain phantom***

According to Borghammer et al. (9, 11), lower activity and metabolism is seen in the grey matter of patients with neurodegenerative diseases; these changes are less likely to occur in white matter regions, such as the cerebellum and pons. Based on such reports, we prepared a mask and applied it to the data from 11 healthy subjects to construct a digital brain phantom in which metabolism and activity were decreased in the grey matter, while the white matter regions, the cerebellum, and pons, showed no changes. For the attenuation rate, we prepared the digital brain phantom based on the reports of Borghammer et al. (9, 11,19, 20) and compared count normalizations. 

***Three-dimensional extraction of brain data***

Using the digital brain phantom function of the Prominence Processor (21), we extracted 3 three-dimensional patterns of brain data: whole brain, cerebral grey matter excluding the cerebellum and pons, and cerebral grey matter of the left occipital lobe. The Prominence Processor, which have 63 various applications classified into 6 groups, is a major software package for education and research in the field of nuclear medicine in Japan. One of the Prominence Processor function is to create arbitrary digital brain phantom of the analyze data format which is appropriated to SPM analysis.

***Anatomic normalization***

To prepare a digital brain phantom based on data from the healthy subjects and attenuation obtained using a mask, both sets of data must have the same form. However, the data from the cerebral grey matter and the grey matter of the left occipital lobe do not have the same form as data from the whole brain. As a result, accurate anatomic normalization was not possible. Therefore, data from the cerebral grey matter and the grey matter of the left occipital lobe were converted to the same form of data as the whole brain. This was done by adding the whole brain data to data from the cerebral grey matter and the grey matter of the left occipital lobe using MATLAB as a pretreatment to anatomic normalization. We performed anatomic normalization of the FDG-PET data collected from the 11 healthy subjects on whole brain data, combining whole brain and cerebral grey matter, and on combined data from the whole brain and the grey matter of the left occipital lobe. This was done by using an anatomical standard brain, which is an FDG-PET template built into SPM8.

***Preparation of the mask***

1) Slight-decrease mask: We obtained data for the cerebral grey matter only by subtracting the whole brain data from combined data from the whole brain and the cerebral grey matter. We prepared the slight-decrease mask by adding an 11% attenuation rate. 

2) Intermediate-decrease mask: We obtained data for the grey matter of the left occipital lobe only by subtracting the whole brain data from combined data from the whole brain and the grey matter of the left occipital lobe. We obtained the area of slight decrease in the intermediate-decrease mask by removing the area of the grey matter of the left occipital lobe from the area of the cerebral grey matter. To create the intermediate-decrease mask phantom, attenuation rates of 11% and 23% were applied to the areas of the cerebral grey matter with slight decrease and the grey matter of the left occipital lobe, respectively.

***Preparation of slight-decrease and intermediate-decrease digital brain phantoms***

**Figure 1:**
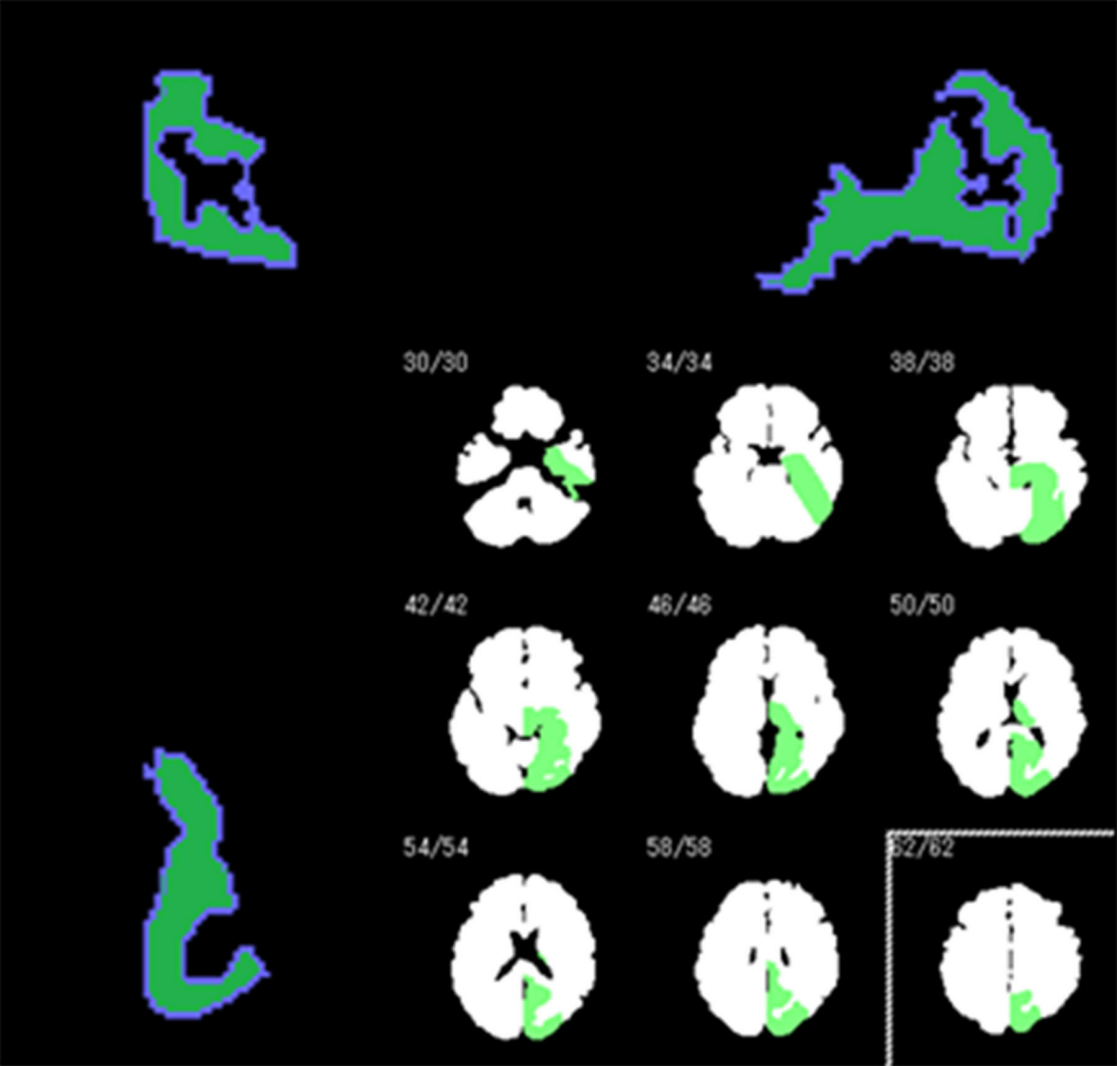
The mask used to prepare a digital brain phantom that mimics an intermediate neurodegenerative disease by adding decreased metabolism to the data of healthy subjects. Decreased metabolism is shown in the cerebral grey matter of the left occipital lobe (green area in the bottom right image)

We applied the slight-decrease and intermediate-decrease masks, to FDG-PET data collected from the 11 healthy subjects, which had undergone anatomic normalization using SPM8. We smoothed the data for a FWHM of 10 mm using SPM8. We then prepared 11 slight-decrease digital brain phantoms and 11 intermediate-decrease digital brain phantoms (Figure 1). 

***Comparisons of each count normalization method using simulation data***

***Count normalization methods***

***GM count normalization***

Using MATLAB and the fMRIstat program, we calculated the mean count for the whole brain (excluding the ventricles and cerebrospinal fluid outside of the brain) and performed GM count normalization. 

***VOI count normalization***

We used the total sum for the cerebellum and the pons as the standard for the count normalization. Using MATLAB, we calculated the mean for the anatomical site in each slice. We then summed the means from the cerebellum and the pons for each slice and used this value as the standard for the count normalization.

***Andersson method***

**Figure 2:**
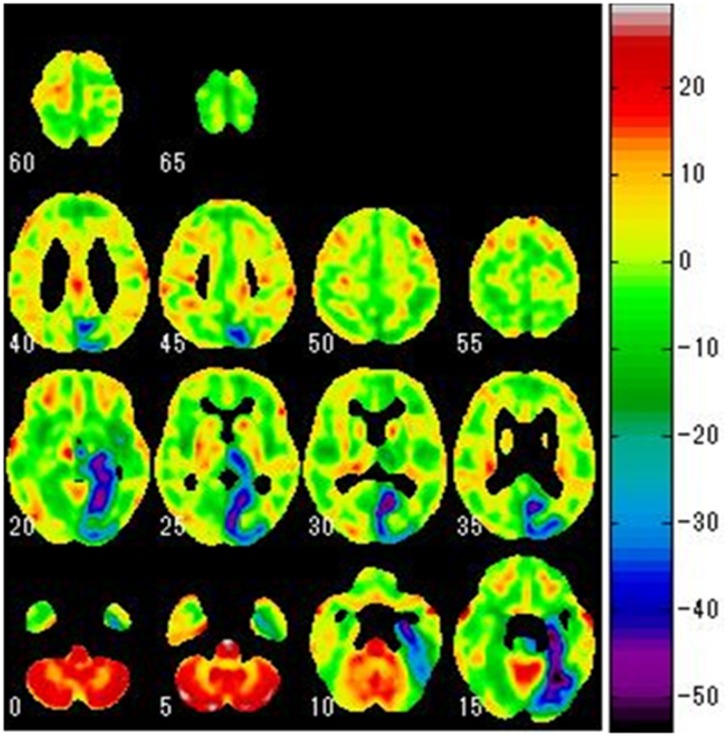
A t-map prepared based on the results of a t-test for each voxel during the SPM analysis process. The image is that of lowered metabolism in the cerebral grey matter of the left occipital lobe

**Figure 3:**
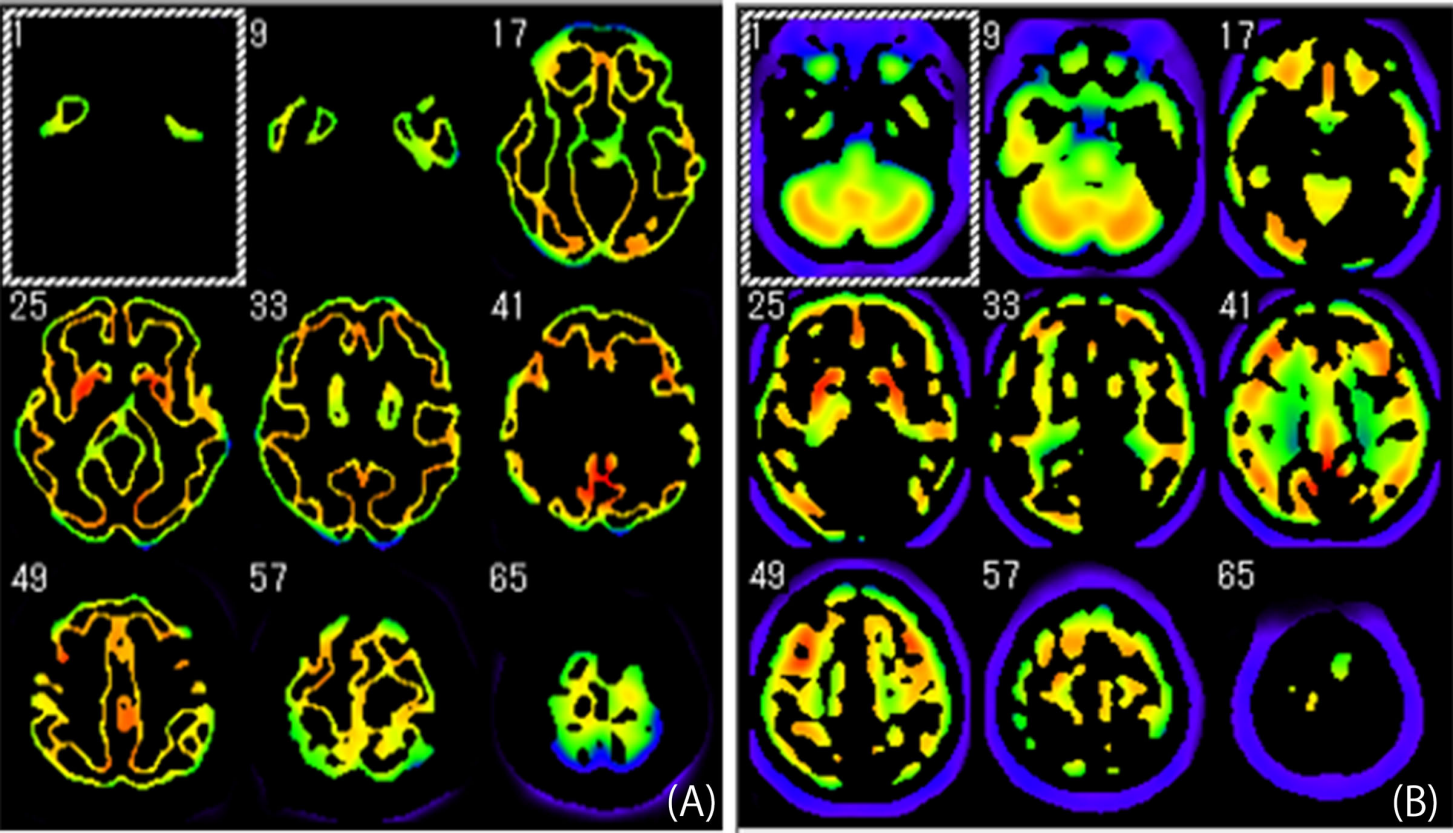
Areas extracted from the data used as the reference areas to calculate the standard for count normalization

1) Using the GM count normalization, the SPM analysis process up to the t-test step was performed on the data. 

2) As a t-test was performed for each voxel, a t- map was prepared in which the results for each voxel were presented (Figure 2). 

3) Using the prepared t-map, the areas with significant changes at any threshold were excluded as we prepared a mask only for the areas with small changes. The range of threshold processing was set to -2 < t < 2, as per the Andersson method. 

4) To extract the reference area, the prepared mask was applied to all of the data (Figure 3A). We calculated the mean count from the reference area extracted from the whole data to obtain the standard value for the count normalization. 

5) Using the calculated standard value, we performed the count normalization on all the data.

6) We processed the data subjected to the count normalization up to the t-test step in the SPM analysis process.

7) We repeated steps (2) through (6) 3 times and performed count normalization using the standard value obtained in the third extraction of the reference area. The result was considered as the final result of the SPM analysis using the Andersson method.

***Yakushev method***

We prepared a t-map in the same manner as in the Andersson method. The range of the extracted reference area for the t-map was t > 2 (p < 0.05), and the extraction was performed once. 

1) We performed the SPM analysis on the data up to the t-test step using GM count normalization. 

2) As the t-test was performed for each voxel, we prepared a t-map for the results of each voxel (Figure 2). 

3) Using the prepared t-map, excluding the areas with significant changes at any threshold, we prepared a mask only for the areas with small changes. The range of threshold processing was set to t > 2 (p < 0.05).

4) To extract the reference area, we applied the prepared mask to all of the data to be analyzed (Figure 3B). To obtain the standard value for the count normalization, we calculated the mean count from the reference area extracted from the whole data.

5) We performed count normalization using the standard value obtained in the extraction of the reference range. The result was considered as the final result of the SPM analysis using the Yakushev method.

***Examination of the impact of the number of extractions in the Andersson method***

While using the Andersson method, we increased the number of reference area extractions to 20 in an increment manner and compared the results to those obtained by running the reference area extraction 3 times.

***Examination of the threshold for reference area extraction in the Yakushev method***

While using the Yakushev method, we increased the threshold of the reference area in steps and compared the resulting data.

***Statistical analysis***

We used MATLAB and fMRIstat provided free of charge by Worsley et al. for our statistical analyses. fMRIstat allows for analyses nearly identical to those commonly performed using SPM. We used the mixed effects model proposed in chapter four of the paper by Worsley et al. (22) our analytical model and increased the degree of freedom by smoothing the standard deviation image. In addition, we obtained the t-statistics value for each voxel in the brain using the 3D Gaussian Random Field Theory (23) and applied a threshold (p < 0.05) to the obtained t-statistics. We then plotted the results onto the image of the brain. 

## Results

***The slight-decrease phantom simulation***

**Figure 4:**
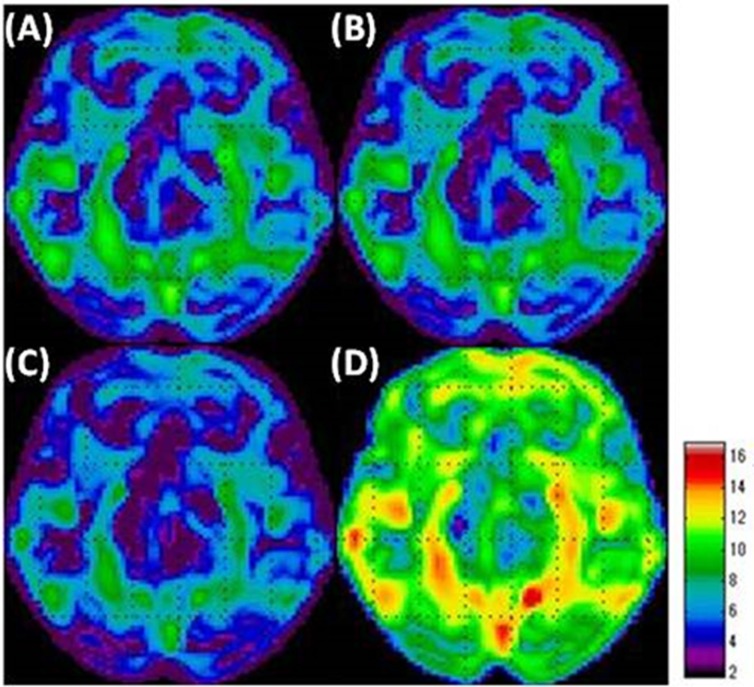
Simulation results of the area of decreased metabolism in the “slight-decrease phantom” in SPM analyses using each of the count normalization methods.

**Figure 5:**
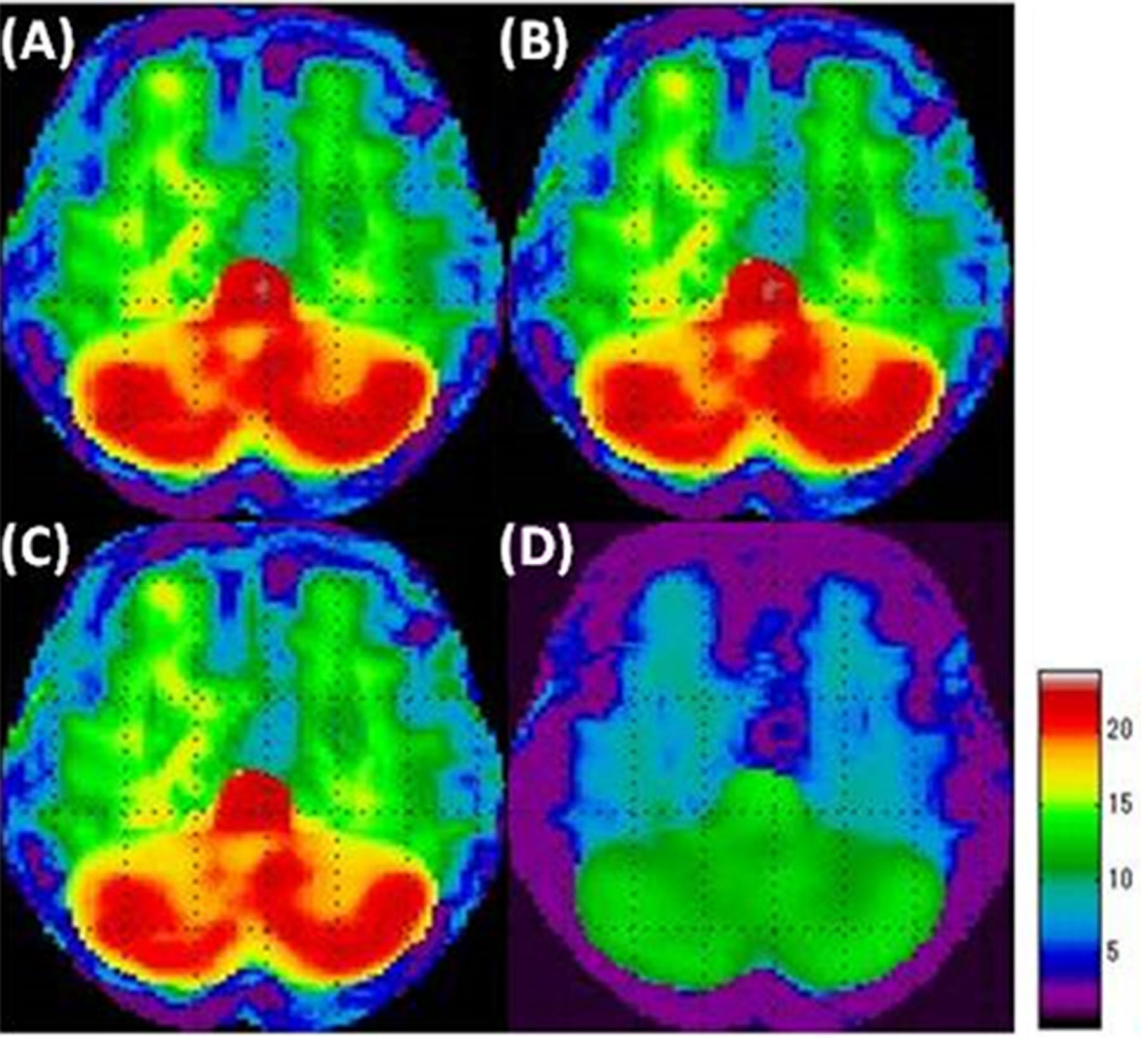
Simulation results of the area of increased metabolism in the “slight-decrease phantom” in SPM analyses

When SPM analysis was performed on the slight-decrease phantom simulation using the GM count normalization (Figure 4A), the VOI count normalization (Figure 4B), and the Andersson method (Figure 4C), we were unable to identify the area with slight-decreased metabolism in the grey matter, which is the area with a slight decrease. Thus, our overall understanding was incomplete using these methods. Specifically, using the Andersson method led to a small captured area. Using the Yakushev method (Figure 4D), we could visualize a wider area of decreased metabolism compared with the other methods. Yakushev method especially captured the decreases in the grey matter and successfully indicated as the decreases in the digital brain phantom. GM count normalization (Figure 5A), VOI count normalization (Figure 5B), and Andersson method (Figure 5C) generally led to artifacts of increased metabolism, in areas without decreased function, strongly in the cerebellum. The Yakushev method (Figure 5D) had notably fewer artifacts than the other count normalization methods.

***The intermediate-decrease phantom simulation***

**Figure 6:**
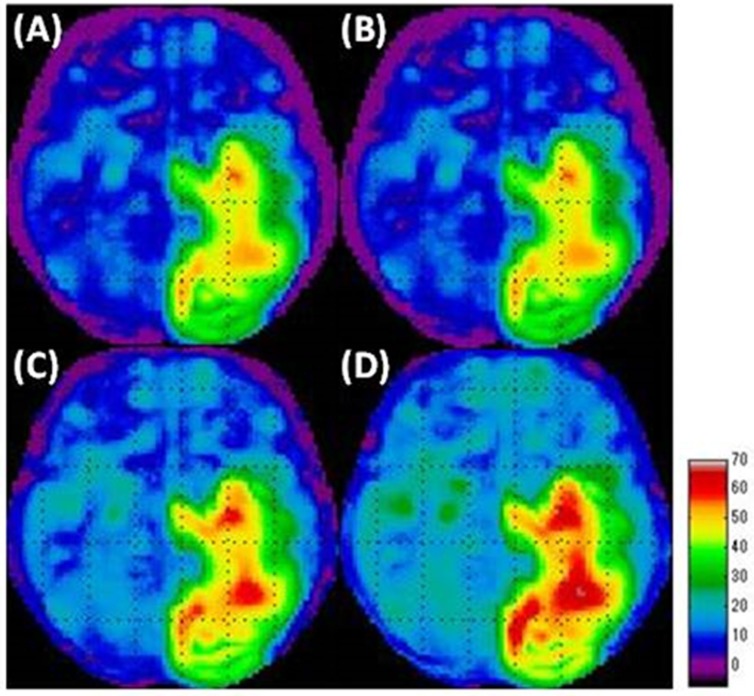
Simulation results of the area of decreased metabolism in the “intermediate-decrease phantom” in SPM analyses

The areas with intermediate decreases were generally well visualized with all the count normalization methods (Figure 6). The areas with slight decreases were not visualized when using GM (Figure 6A) and VOI count normalizations (Figure 6B). The Andersson method (Figure 6C) resulted in wider areas of slight decreases compared to GM and VOI count normalizations. The areas with slight decreases were well visualized with the use of Yakushev method (Figure 6D).

**Figure 7:**
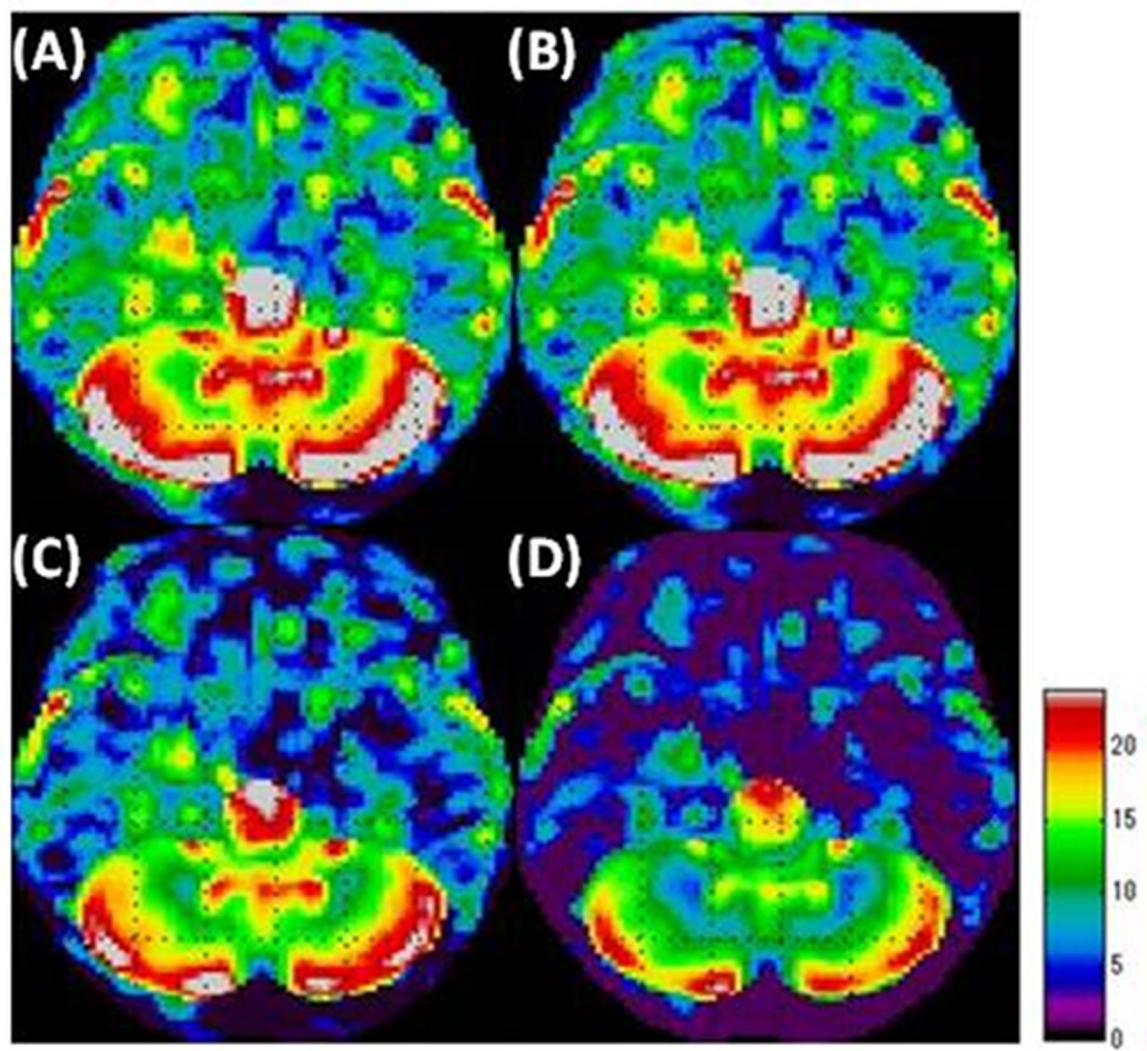
Simulation results of the area of increased metabolism in the “intermediate-decrease phantom” in SPM analyses

We observed artifacts of hypermetabolism in all the count normalization methods (Figure 7). GM (Figure 7A) and VOI count normalizations (Figure 7B) led to prominent artifacts, especially in the cerebellum. When using the Andersson method (Figure 7C), the area of these artifacts was small compared to that obtained by using GM and VOI count normalizations. The Yakushev method (Figure 7D) led to relatively fewer artifacts.

***Impact of the number of extractions in the Andersson method***

When the number of extractions for the reference area was increased, the decreased metabolism area in the grey matter of the digital brain phantom was better captured compared to when three extractions were used. In addition, we were able to capture the artifacts of increased metabolism by increasing the number of reference area extractions. 

(A) Andersson method

(B) Yakushev method

(A) GM count normalization

(B) VOI count normalization

(C) Andersson method

(D) Yakushev method

(A) GM count normalization

(B) VOI count normalization

(C) Andersson method

(D) Yakushev method

(A) GM count normalization

(B) VOI count normalization

(C) Andersson method

(D) Yakushev method

(A) GM count normalization

(B) VOI count normalization

(C) Andersson method

(D) Yakushev method

***Examination of the threshold for reference area extraction in the Yakushev method***

Raising the threshold for the reference are extraction in the Yakushev method did not lead to any significant changes in the visualization of the area with decreased metabolism. However, increasing the threshold for the reference area extraction reduced the artifacts of increased metabolism. 

## Discussion

***GM count normalization***

When performing SPM analysis using GM count normalization in neurodegenerative disease, it is important to consider the possibility of a wider area with decreased metabolism than that indicated by the results. It is also important to keep in mind that areas without increased metabolism may be visualized as areas of increases when analyzing the results. Here we report that performing an SPM analysis using GM count normalization when evaluating a neurodegenerative disease leads to the incomplete capture of the area with decreased metabolism. Furthermore, this method leads to the frequent visualization of areas of increased metabolism in the healthy areas, which are likely artifacts. This supports the report by Borghammer et al. (20). If the status of the brain is evaluated using an SPM analysis with GM count normalization, only the areas with significant decreases in metabolism may be visualized as the areas of decrease, while areas with slight decreases may not be visualized. Therefore, this method is likely to underestimate the areas with decreased metabolism. This does not mean that the areas of decrease visualized in an SPM analysis using GM count normalization are incorrect, as these are definitely the areas of decreased metabolism. However, areas with slight decreases may not be captured by this method due to the use of areas that are altered by disease as the reference range. Increases in metabolism, which are likely artifacts, were found in wider areas in the intermediate-decrease digital brain phantom (Figure 7) than in the slight-decrease digital brain phantom (Figure 5). In other words, stronger artifacts were found in cases with higher clinical severity. This is because in severe cases, the decrease in metabolism is more significant, which leads to a further decrease in the standard value for GM count normalization, which in turn emphasizes the artifact of increased metabolism. This is thus an issue when using GM count normalization for the analysis of severe cases. 

***VOI count normalization***

The results of SPM analysis using VOI count normalization on the digital brain phantom were similar to that of GM count normalization. Therefore, caution must be taken to use this method in evaluating neurodegenerative disease.

***Andersson method***

Unlike GM count normalization, the Andersson method (14) could not capture the decreased metabolism area in the digital brain phantom. In addition, there was an increased metabolism area, which is likely an artifact. The decreased area in the slight-decrease phantom had a narrow visualization (Figure 4C). However, increasing the number of reference area extractions led to the visualization of a wider area with decreased metabolism and reduced the artifact of increased metabolism. In other words, it could be a highly accurate count normalization method if the number of reference area extractions is increased. We observed an artifact of the areas without decreased metabolism as areas with increased metabolism, which is a different result that reported by Borghammer et al. (20). One explanation for this finding may be that the digital brain phantom prepared in this study had a wider area of decreased metabolism compared to the one used by Borghammer et al. (11,19, 20). The wider area of decreased metabolism corresponds to higher clinical severity. Thus, if SPM analysis is performed with this method on a neurodegenerative disease with high severity, the number of reference area extractions should be adjusted depending on the severity of the disease. In addition, while we examined the effects of changing the number of processing steps, it will be necessary to examine the width of the threshold in future studies, as it was fixed at -2 < t < 2 in the present study. It may be possible to obtain more accurate results by changing the threshold at each step of the process, which is something that should be further examined.

***Yakushev method***

The Yakushev method is the most suitable count normalization method for the SPM analysis of neurodegenerative diseases among all the count normalization methods examined in this study. Compared to other methods, the area of the slight decrease in metabolism could be visualized more accurately as a wide area. It resulted in the smallest area of the artifacts of increased metabolism in healthy areas*. *Therefore, this method was considered to be the most favorable method to use for areas with both increased and decreased metabolism. However, it still led to artifacts of increased metabolism. As discussed for the Andersson method, the digital brain phantom prepared in this study had a wider area of decreased metabolism than that used in the report by Borghammer et al. (11, 19, 20). This may explain the presence of the above artifact. The Yakushev method consists of first performing the GM count normalization and presenting the results from the subsequent t-test as a t-map. Based on the obtained t-map, the reference area is extracted after threshold processing. The standard value for the GM count normalization decreases as the ratio of the area with decreased metabolism in the brain increases. Since this method leads to a lower standard value*, *the count normalization is performed at a value lower than normal and the number of counts for each voxel decreases overall. The t-map is prepared for the data that have been count normalized using the lower standard value and threshold processing is performed at the constant value of t > 2 (p < 0.05). This leads to a lower value for the reference area used, lowering the standard value. Thus, the standard value for the reference area obtained in the extraction process decreases. Here, we reduced this artifact by increasing the threshold, which allowed us to accurately capture the area of decreased metabolism. These results indicate that the threshold must be set according to the severity of the disease, and that future examination of this point is necessary. 

Here we report that the Yakushev method is the most suitable count normalization method when metabolism is decreased in the whole brain. In other words, the Yakushev method is suitable to analyze patients with diseases such as neurodegenerative diseases. However, it may not be ideal if the decrease in metabolism is localized. The Yakushev method assumes that artifacts of increased metabolism area created the standard value decreases in the initial GM count normalization*. *If the area with decreased metabolism is highly localized, the initial GM count normalization will offset the decrease in metabolism, preventing the decrease in the standard value. Therefore, the threshold in the Yakushev method, which defines the area of increased metabolism as the reference area, may not be suitable. Thus, the Yakushev method is not suitable to analyze all neurodegenerative disease. Future studies are necessary to resolve this issue.

Further studies are needed in order to perceive the comparison between healthy subjects and patients with Alzheimer’s disease using SPM analysis with GM count normalization, VOI count normalization, the Yakushev method and the Andersson method. The two groups’ comparisons are now considering in our laboratory. We will report about the comparison of the normalization methods with the patients in a future work.

## Conclusion

When analyzing the neurodegenerative disease with SPM analysis, it is extremely difficult to fully describe the status of the brain. It is important to review the results after processing using different modalities. If there are differences in the obtained results, the most suitable count normalization may need to be selected based on the results from several different count normalization methods. 

The present study’s evaluations were based only on a digital brain phantom. However, we ultimately aim to select the ideal count normalization method to use with data from a real diseased brain to determine whether the similar results can be obtained with the actual patients. 

We compared count normalization methods for the analysis of digital brain phantoms meant to resemble brains with the neurodegenerative disease condition. The Yakushev method was shown to be the most desirable count normalization method for the SPM analysis of diseases, such as neurodegenerative disease in which metabolism is decreased throughout the brain. The GM count normalization may underestimate the areas with decreased metabolism and lead to artifacts of increased metabolism. In severe cases, the Yakushev method and the Andersson method may need to be altered to obtain more accurate analyses by raising the threshold for the reference area extraction process and increasing the number of the reference area extractions, respectively.
